# Granulosa Cell Specific Loss of Adar in Mice Delays Ovulation, Oocyte Maturation and Leads to Infertility

**DOI:** 10.3390/ijms232214001

**Published:** 2022-11-13

**Authors:** Rikki N. Nelson, V. Praveen Chakravarthi, Anamika Ratri, Xiaoman Hong, Jan A. Gossen, Lane K. Christenson

**Affiliations:** 1 Department of Cell Biology and Physiology, University of Kansas Medical Center, Kansas City, KS 66160, USA; 2 Osteo-Pharma BV, 5349 AB Oss, North Brabant, The Netherlands

**Keywords:** granulosa cell, fertility, ovulation, ADAR RNA editing, post transcriptional regulation, periovulatory follicle, transcriptome

## Abstract

Adenosine deaminases acting on RNA-(ADAR) comprise one family of RNA editing enzymes that specifically catalyze adenosine to inosine (A-to-I) editing. A granulosa cell (GC) specific *Adar* depleted mouse model [*Adar ^flox/flox^*:*Cyp19a1-*Cre/+ (gc*Adar*KO)] was used to evaluate the role of ADAR1 during the periovulatory period. Loss of *Adar* in GCs led to failure to ovulate at 16 h post-hCG, delayed oocyte germinal vesicle breakdown and severe infertility. RNAseq analysis of GC collected from gc*Adar*KO and littermate control mice at 0 and 4 h post-hCG following a super-ovulatory dose of eCG (48 h), revealed minimal differences after eCG treatment alone (0 h), consistent with normal folliculogenesis observed histologically and uterine estrogenic responses. In contrast, 300 differential expressed genes (DEGs; >1.5-fold change and FDRP < 0.1) were altered at 4 h post-hCG. Ingenuity pathway analysis identified many downstream targets of estrogen and progesterone pathways, while multiple genes involved in inflammatory responses were upregulated in the gc*Adar*KO GCs. Temporal expression analysis of GCs at 0, 4, 8, and 12 h post-hCG of *Ifi44*, *Ifit1*, *Ifit3b*, and *Oas1g* and *Ovgp1* confirmed upregulation of these inflammatory and interferon genes and downregulation of *Ovgp1* a glycoprotein involved in oocyte zona pellucida stability. Thus, loss of ADAR1 in GCs leads to increased expression of inflammatory and interferon response genes which are temporally linked to ovulation failure, alterations in oocyte developmental progression and infertility.

## 1. Introduction

Ovulation or release of a fertilizable egg from the large preovulatory follicle represents the culmination of follicular growth and onset of corpus luteum formation. Disruption of ovulation is a major cause of infertility as well as a prime target for many contraceptive mechanisms, as such the transcriptional, post-transcriptional and cell-signaling events that occur within the ovary following the ovulatory surge of LH has garnered much research [1,2]. Uniquely, post-transcriptional gene regulatory events have primarily focused on microRNA regulation within the ovarian somatic cells, while in the oocyte all aspects from splicing to translation (i.e., the boundaries of post-transcriptional gene regulation) have been extensively studied in the transcriptionally quiescent germinal vesicle oocyte [3]. 

Post-transcriptional gene regulatory studies have revealed a ubiquity of mRNA modifications from deposition of chemical moieties as seen in *N*6-methyladenosine to complete catalysis of transition as seen in adenosine to inosine (A-to-I) editing [4]. In the case of A-to-I editing, three members of the adenosine deaminases acting on dsRNA (ADARs) enzyme family exist, with *Adar* (ADAR1) and *Adarb1* (ADAR2) being catalytically active, while *Adarb2* (ADAR3) does not catalyze the A-to-I transition [5]. All ADARs maintain the dsRNA binding domain, thus preserving the potential of *ADAR3,* in addition to ADAR1 and ADAR2, to elicit effects through non-canonical mechanisms [6]. Additionally, *Adar* generates two isoforms; a small constitutively expressed p110 isoform generally found in the nucleus, and a larger interferon-induced cytoplasmic p150 isoform [7]. The functional implications of ADARs at the molecular level, includes alterations of coding sequences, modification of alternative splicing acceptor sites, changes of 5’ and 3’ untranslated regions, including modification of miRNA biogenesis to target recognition sites [8,9], as well as in the ability to sequester transcripts from degradation [10]. Effects of ADARs are ubiquitous, however ADAR2 is commonly considered important primarily in the brain, while ADAR1 (*Adar*) is more strongly expressed in most other tissues [11]. To identify A-to-I edits, RNA seq results are compared to DNA seq results and guanines in locations where adenosine should be present represent putative deamination events as inosines are recognized by reverse transcriptase as guanine residues [12,13]. 

The status of A-to-I editing as the most prevalent mammalian RNA editing event [11,14,15], begs the question of its physiological role. However, organism-wide *Adar* deletion in mice leads to embryonic lethality due to liver disintegration and hematopoietic insufficiency, preventing investigation in adult tissues [16]. Implementing models for spatially and temporally controlled *Adar* deletion revealed ADAR1’s critical role in inducing organ fibrosis because of decreased cell proliferation and survival in the murine liver and the adult heart [17,18]. ADAR1 is proposed to protect against stress-induced apoptosis in developing vertebrae, heart, liver, and mouse embryonic fibroblasts [19]. Exploration of ADAR1’s role in the female reproductive system as another highly proliferative tissue, where inflammation is closely associated with the ovulatory process [20,21] has not been undertaken. A-to-I editing occurs in mammalian (human, porcine, and murine) ovarian tissues [22,23,24,25,26]. In fact, ovarian and testicular tissue exhibited the greatest levels of A-to-I edits of all tissues in zebrafish [24] and in the pig, the ovary exhibited the second highest level of editing for a tissue after the brain [22,24]. Whole organ studies of a dynamic organ, such as the ovary, however, leaves many unresolved questions, including the identities of the cell types within the ovary where A-to-I editing occurs, whether *Adar* expression is hormonally regulated and lastly its functional role. Due to the unique presence of germ cells, in the ovary and testis, it is easy to speculate that oocytes may account for the elevated A-to-I editing seen in ovarian tissue. Indeed, our laboratory and others demonstrated that mouse oocytes collected at germinal vesicle (GV), and metaphase II (MII) stages displayed abundant A-to-I RNA editing [13,25,26,27] and ADAR1 detection by Western blot in as few as 10-20 oocytes [13] supports this premise. The role of ADAR1 in the ovarian somatic cells particularly the highly proliferative follicular granulosa cell which are critical for all aspects of ovarian function including folliculogenesis, ovulation, and formation of the corpus luteum need to be studied to know whether ADAR1 has any role in these tissue events. 

ADAR1’s role in mural granulosa cells (mGCs) function of the periovulatory follicle will be examined by targeted deletion of the gene in mGC at the transition from pre-antral to an antral follicle (i.e., onset of *Cyp19A1* expression). Detailed molecular, tissue and whole animal functional assessments of the conditional KO and littermate control, will address ADAR1’s role in the female reproductive system and fertility. 

## 2. Results

### 2.1. Conditional Deletion of Adar in mGC Renders Female Mice Infertile

**Figure 1:**
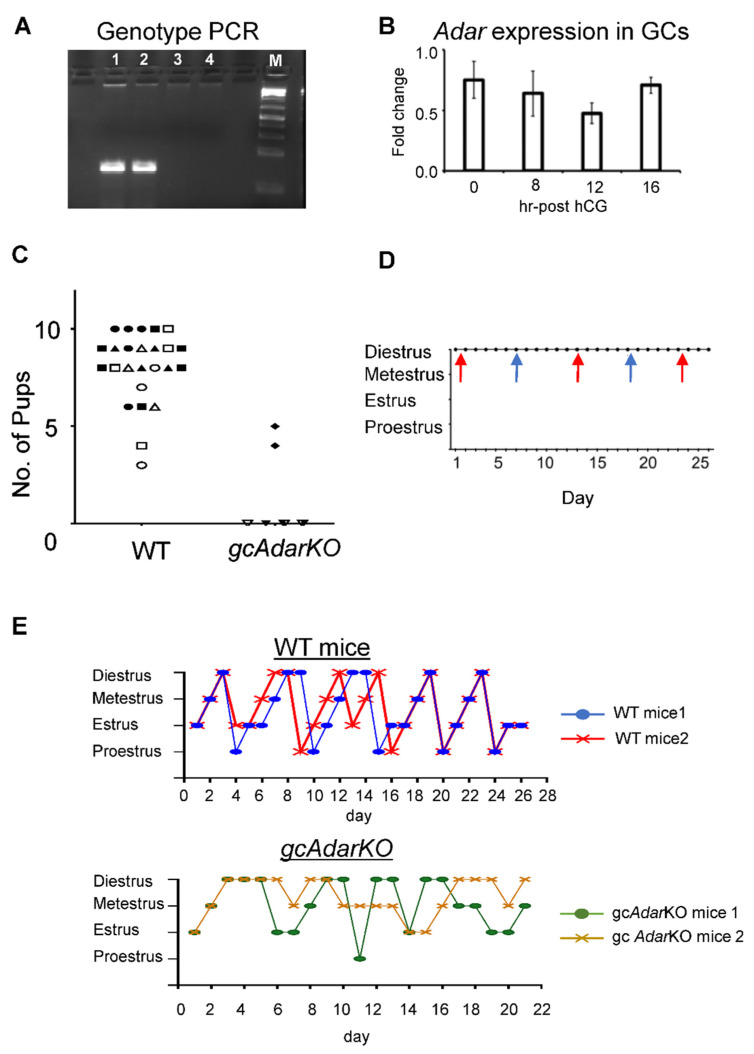
**Granulosa specific *Adar* knockout leads to infertility** (**A**) Genotyping PCR was performed using *Adar* deletion specific primers in granulosa cells from control (lanes 1 and 2) and gc*Adar*KO mice (lanes 3 and 4). ‘M’ represents DNA ladder. (**B**) *Adar* expression in ovarian granulosa cells at different time points post-hCG was determined using RT-qPCR. The expression was normalized with 18S rRNA. (**C**) Pups per litter from wild-type littermate control mice (n = 6) and gc*Adar*KO (n = 5) were counted over a 7-month period. Individual mice are indicated by unique icons. Each litter is plotted by the number of pups counted or placed at zero to indicate no litters were ever observed. (**D**) Estrous stage for representative gc*Adar*KO breeding trial mice as determined by vaginal cytology, presence of seminal plugs is indicated by arrows, (different colors represent individual animals, n = 2 shown of the 5). (**E**) Estrous cycle stages for representative wild-type and gc*Adar*KO females analyzed by vaginal smears collected from 42-day old mice for at least 21 days. Smears were stained with crystal violet staining and staged based upon cytology. Representative graphs for 2 WT and 2 gc*Adar*KO are shown. Wild-type mice displayed cyclical estrous patterns, while gc*Adar*KO had disrupted cycling with prolonged periods of diestrus and metestrus.

Granulosa specific *Adar* KO was confirmed by genotyping using deletion specific primers *in* gc*Adar*KO and WT (littermate control) mice mGC (Figure 1A). Adar expression did not vary in mGC following hCG (Figure 1B). Control female littermates (*Adar ^flox/flox^* or *^flox/+,^ lacking the Cyp19a1*-Cre) all produced at least three litters each with an average of 8.0 ± 0.4 pups per litter, while 4 of 5 gc*Adar*KO female mice failed to produce any offspring over a 7-month breeding trial, while the single gc*Adar*KO female that did deliver pups, had two litters of four and five pups and no further litters (Figure 1C). At 5 months of the breeding trial, vaginal cytology and breeding was confirmed by visualization of a seminal plug or presence of sperm in vaginal smears over a 26-d period in gc*Adar*KO females. Plugs or sperm were found every 10–12 days (Figure 1D). Cytology indicative of pseudopregnancy was observed in each smear between breeding events in the breeding trial animals. At the end of breeding trial, female mice were euthanized, and gross reproductive tract morphology appeared normal with uterine horns appearing normal in width and length as were ovaries.

### 2.2. Estrous Cyclicity Disturbances in gcAdarKO Mice

Estrous cycle staging of WT (control littermates) mice progressed chronologically as expected, from proestrus, to estrus, metestrus and diestrus before repeating, while gc*Adar*KO exhibited erratic estrous cycle patterns, with prolonged periods of metestrus and diestrus observed. Proestrus was almost never identified (Figure 1E).

### 2.3. Histological Analysis of Ovaries

**Figure 2:**
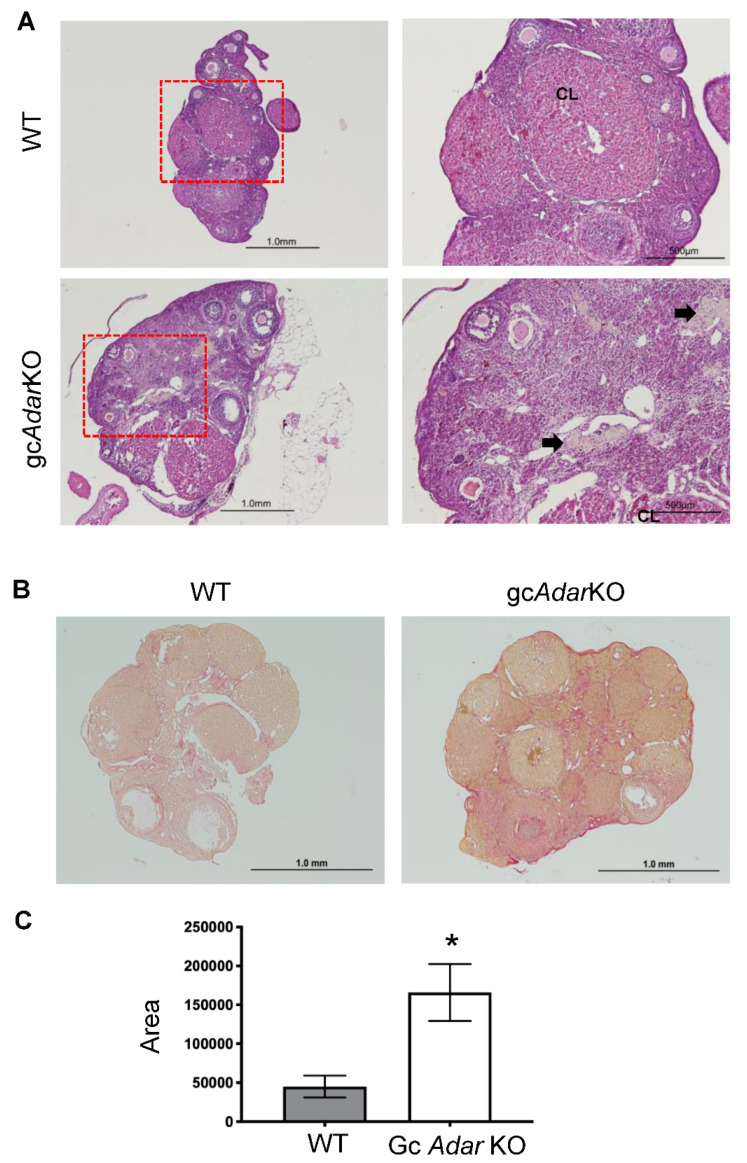
**Histological analyses.** (**A**) Histology analyses was performed in the ovaries from wild-type (WT) littermate control and gc*Adar*KO (n = 5) breeding trial mice (n = 6). Sections from the middle region of tissue were selected for H&E staining. Control ovaries display corpora lutea (CL) and follicles of varying size. Ovaries from gc*Adar*KO mice display fewer corpora lutea and similar follicle sizes. Yellow-multinucleated (arrows) cells can be seen in all gc*Adar*KO ovaries. (**B**) Picrosirius red staining was completed in the ovaries of WT and gc*Adar*KO mice. (**C**) Wild-type mice (n = 3) exhibited red staining intensity of 45,133 ± 14,019 pixels/µm^2^, while gc*Adar*KO mice (n = 4) had increased (*p* = 0.04) intensity at 166,005 ± 36,643 pixels/µm^2^. * Denotes significant (*p*<0.05) difference between means ± SEM.

Ovaries collected from gc*Adar*KO and WT (littermate control) mice at the completion of 7 months breeding trial exhibited follicles of various sizes in central sections of ovarian tissue in both genotypes (Figure 2A). A preponderance of multinucleated yellow to brown cells were visualized in the stroma of the ovarian tissue of the gc*Adar*KO females, and picrosirius red staining (PSR) revealed an increase in extracellular matrix deposition, presumed to be collagen, in the gc*Adar*KO ovaries when compared to the breeding trial WT ovarian tissue (Figure 2B,C). In order to ensure this difference was not due to the intervening pregnancies that occurred only in the WT females, ovarian PSR staining was compared in another experimental trial using virgin aged WT and gc*Adar*KO females. No significant differences in extracellular matrix deposition were found across a 16-month analysis of aged WT animals or in the gc*Adar*KO (2–16 m) females (Supplementary Figure S1).

### 2.4. Delayed Ovulation in gcAdarKO Mice

**Figure 3:**
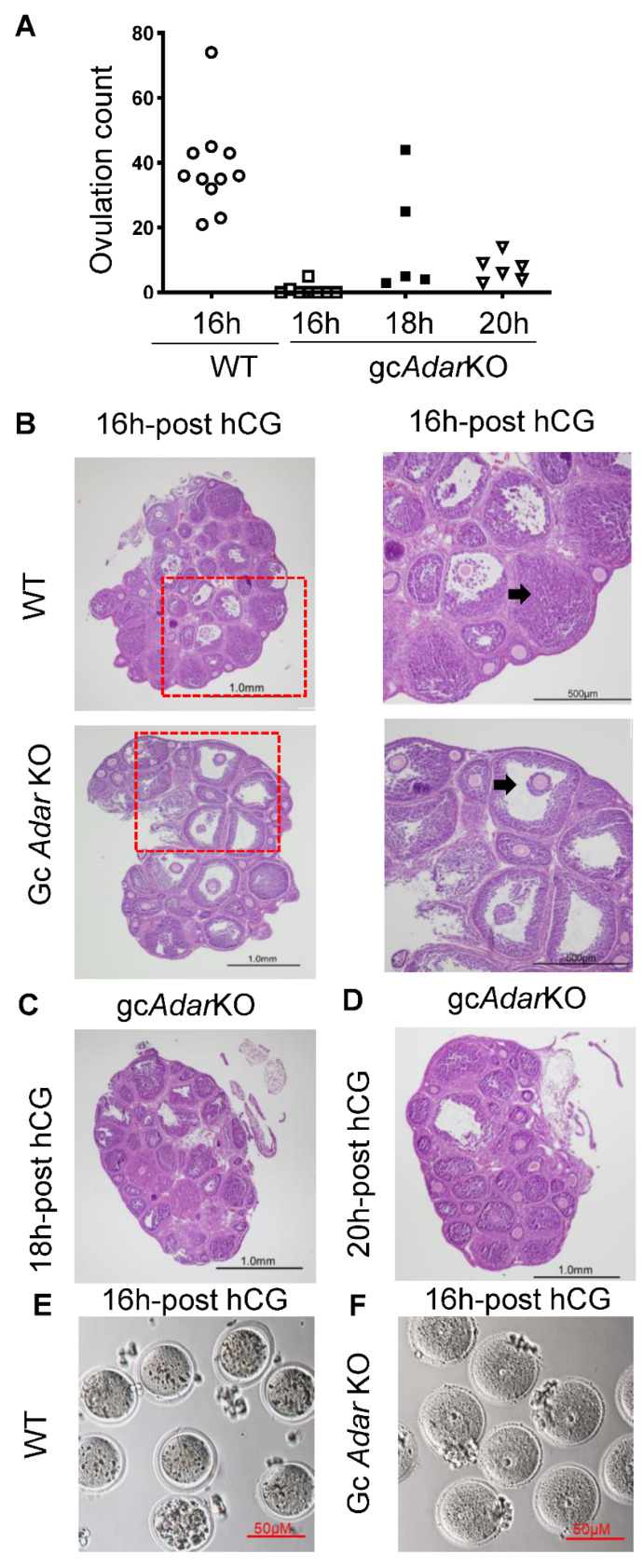
**Granulosa specific *Adar* knockout leads to delayed and altered ovulation.** (**A**) Ovulated eggs retrieved from oviducts 16, 18, and 20 h after ovulation induction. Eggs were collected from oviducts of immature, 24-day-old WT and gc*Adar*KO after 46 h of eCG stimulation followed by 16, 18 and 20 h of hCG treatment. A delay and reduction in the number of eggs ovulated was observed in gc*Adar*KO mice compared to WT controls. (**B**) Histological analyses of ovaries from immature superovulated WT and gc*Adar*KO mice. Ovaries from WT mice display corpora lutea (CL) and ovulation sites, while gc*Adar*KO ovaries display trapped oocytes with lack of luteinization of follicles at 16 h post-hCG administration. (**C**, **D**) Variation in luteinized follicles with trapped oocytes (arrowhead) are observed at 18 and 20 h post-hCG administration. (**E**) Ovulated oocytes from WT mice 16 h post-hCG isolated from ampulla of the oviduct. (**F**) Oocytes from gc*Adar*KO ovaries, isolated by puncturing antral follicles with 28-gauge syringe.

Superovulation of immature (21-day-old) WT females ovulated more (*p* < 0.05; 44.0 ± 8.0 mean ± SEM; n = 11) than gc*Adar*KO mice (0.75 ± 0.62; n = 8) at 16 h post-hCG. However, when collection was completed at 18 h or 20 h following hCG administration, eggs were recovered (16.02 ± 8.06; n = 5 and 7.3 ± 1.6; n = 6, respectively) in gc*Adar*KO females (Figure 3A). Eggs from WT mice were released as one large, cloud-like mass of multiple COCs from the oviduct, while eggs from gc*Adar*KO mice were released as individual COCs. As expected at 16 h post-hCG, control ovaries displayed eosinophilic corpora lutea and clear evidence of follicle rupture (Figure 3B), while gc*Adar*KO ovaries follicles did not display the normal evidence of luteinization, and numerous trapped oocytes with densely compacted cumulus cells were observed (Figure 3B). At 16 h post-hCG, oocytes isolated from aspirated follicles of gc*Adar*KO mice showed intact germinal vesicle, while the ovulated oocytes from WT mice showed germinal vesicle breakdown (Figure 3E,F). At 18 and 20 h post-hCG, trapped oocytes were observed in adjacent luteinized follicles and non-luteinized follicles in gc*Adar*KO ovaries (Figure 3C,D).

### 2.5. Transcriptome Analysis of mGCs from gcAdarKO Mice

**Figure 4:**
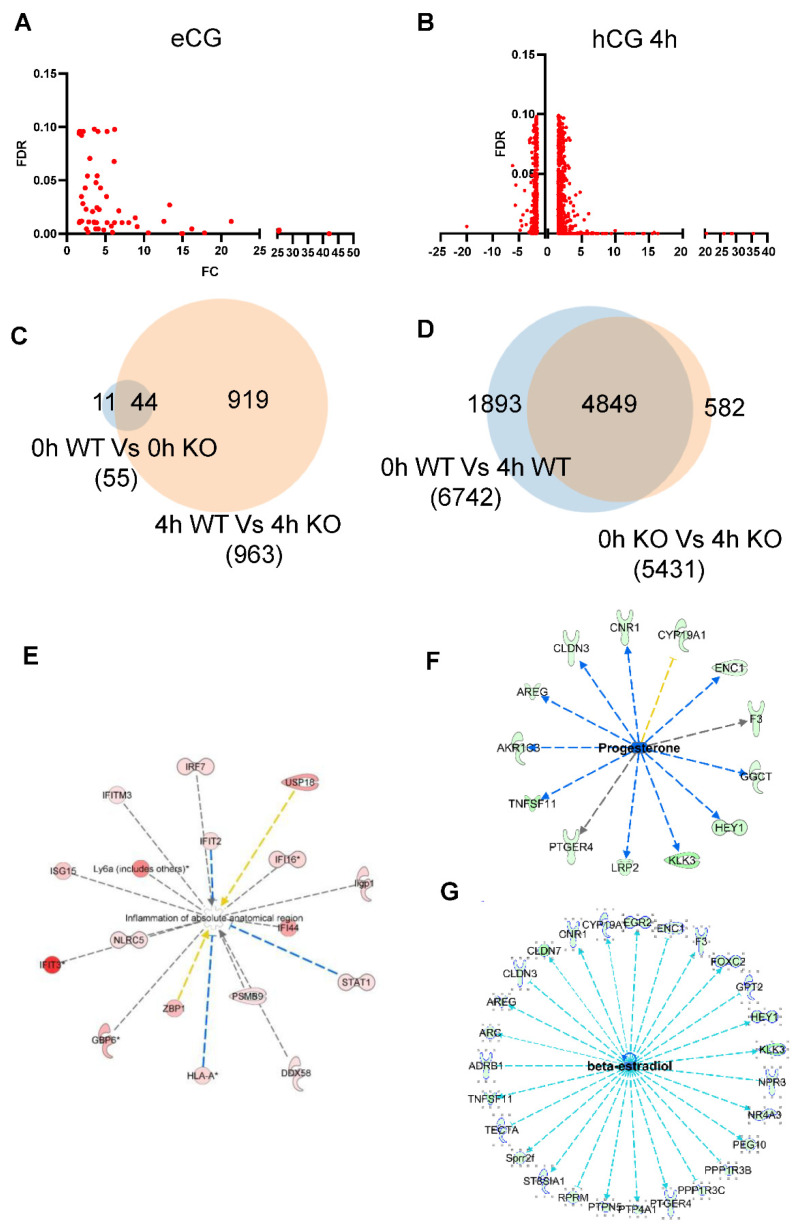
**RNA seq analyses of gc*Adar*KO granulosa cells.** (**A**) Volcano plot showing DEGs with fold change ≥1.5, FDR ≤ 0.1, between WT versus gc*Adar*KO granulosa cells 46 h after eCG injection. Each dot represents a gene. Total there are only 55 upregulated genes. (**B**) Volcano plot showing DEGs with fold change ≥1.5, FDRP ≤ 0.1, between WT versus gc*Adar*KO granulosa cells 4h after hCG injection in eCG primed mice. Each dot represents a gene. (**C**) Venn diagram comparing differentially expressed genes (DEGs) between WT versus gc*Adar*KO GCs 46 h after eCG injection and 4 h after hCG injection in eCG primed mice. (**D**) Venn diagram comparing DEGs 46 h after eCG versus 4 h after hCG injection in eCG primed mice in WT and gc*Adar*KO. (**E**–**G**) IPA analysis revealed three major pathways related to ovarian physiology. (**E**) Inflammation pathway, all genes were highly upregulated in gc*Adar*KO mice granulosa cells. * Denotes significant upregulated genes (*p* < 0.05). (**F**) progesterone and (**G**) estradiol downstream signaling pathways were identified and all of these genes were slightly downregulated.

Comparison of mGC gene expression at 0 h (i.e., eCG treatment only) for WT and gc*Adar*KO indicated a total of 55 differentially expressed genes (DEGs) with a fold change ≥ 1.5 and FDRP value < 0.1 (Figure 4A,C), all of which were upregulated. Comparison at 4 h post-hCG showed a total of 300 DEGs with fold change ≥1.5 and FDRP value < 0.1 between the two genotypes, of which 213 were upregulated and 87 downregulated (Figure 4B,C) of these only 31 DEG exhibited a fold change ≥5.

Comparison of DEGs in 0 h (WT versus gc*Adar*KO) and 4 h hCG (WT versus gc*Adar*KO) groups showed 40 commonly upregulated genes (Figure 4C). Comparison of DEGs from 0 h to 4 h hCG across the WT and gc*Adar*KO genotypes showed 4849 common DEGs with fold change ≥1.5 and FDRP value < 0.1 (Figure 4D) and 582 DEGs are specific for gc*Adar*KO. Complete lists of DEG in mGCs from WT and gc*Adar*KO at 0 and 4 h post-hCG treatment are presented in the Supplementary Tables S1 and S2.

### 2.6. Adar Regulated Pathways in mGCs

**Figure 5:**
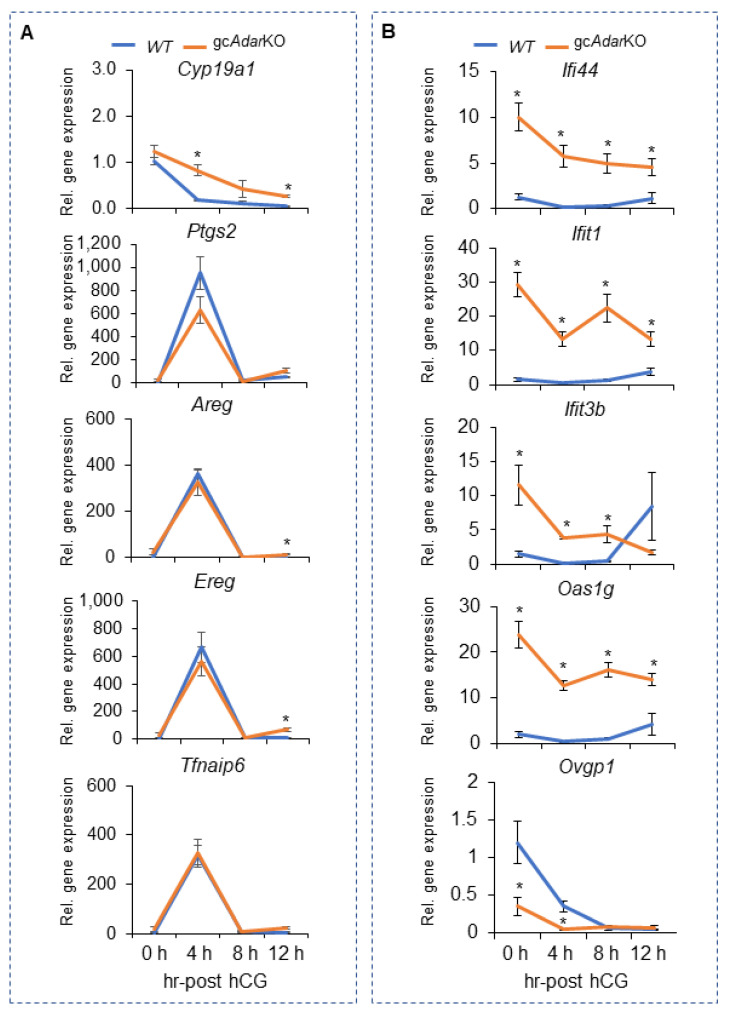
**Temporal expression of known LH regulated genes and of selected genes that exhibited difference following granulosa cell deletion of *Adar*.** Quantitative RT-PCR analysis of (**A**) well-known LH regulated genes (*Cyp19a1, Ptgs2, Areg, Ereg, and Tfnaip6)* and (**B**) select gc*Adar*KO differentially expressed genes (*Ifi44, Ifit1*, *Ifit3b*, *Oas1g and Ovgp1)* in mural GCs from mice (21 days old) collected 46 h after eCG (0 h) or after eCG followed by hCG for 4, 8, 12 h are depicted and shown. qRT-PCR data represent the mean ± SEM n ≥ 6. * Denotes a significant difference (*p* < 0.05) between genotypes.

IPA analysis of the RNA seq data revealed upregulation of several genes related to the inflammatory response pathway (Figure 4E). At the same time genes downstream of progesterone and estradiol signaling were slightly downregulated (Figure 4F,G). The LH induced genes *Ptgs2, Areg, Ereg, and Tfnaip6* all exhibited an almost identical pattern of expression across time for the WT and gc*Adar*KO, while that of *Cyp19a1,* which is down regulated by the LH surge showed a delay in the gc*Adar*KO (Figure 5A). Expression analysis of interferon induced genes *Ifi44*, *Ifit1*, *Ifit3b* and *Oas1g* were confirmed at different time periods of gonadotropin stimulation by qRT-PCR. Expression of *Ifi44*, *Ifit1*, and *Oas1g* genes were significantly greater in gc*Adar*KO compared to WT mGCs (Figure 5B) at all time points. Expression of *Ifit3b* was significantly greater at 0, 4 and 8 h post-hCG but decreased significantly at 12 h in gc*Adar*KO compared to WT mice (Figure 5B). Expression of *Ovgp1* a glycoprotein involved in zona pellucida stability was also decreased significantly at 0 and 4 h post-hCG (Figure 5B).

## 3. Discussion

Granulosa cell specific knockdown of *Adar* guided by *Cyp19a1*-Cre recombinase activity produced female mice with severely diminished fertility. Despite observing positive breeding events, most gc*Adar*KO mice of the breeding trial failed to produce pups. Rhythmic estrous cycling was not observed in gc*Adar*KO mice, indicating a failure in ovarian estrous patterning. While ovulation could be induced in gc*Adar*KO mice, ovulation was significantly delayed as indicated by the failure to detect oocytes in the oviducts of superovulated mice 16 h post-hCG followed by modest recovery rates of oviductal eggs at 18 and 20 h post-hCG. There was a defect in the germinal vesicle breakdown in gc*Adar*KO mice ovaries as observed from the oocytes isolated by puncturing the antral follicles at 16 h post-hCG. Since there was no ovulation 16 h post-hCG in gc*Adar*KO mice, we by necessity collected oocytes from the gc*Adar*KO mice, by aspiration from the large periovulatory follicles. It is important to note that germinal vesicle breakdown occurs at 2-4 h after hCG administration in wildtype mice [28], and we did not observe this in the gc*Adar*KO mice even at 16 h after hCG. Luteinization of the mGC was also delayed as was cumulus expansion. Thus, it appears that gc*Adar*KO females are unable to initiate proper ovulation and differentiation into luteal cells, likely resulting in a dyssynchronous oviductal and uterine environment such that the oocytes that do reach the oviduct are not able to properly implant and maintain a pregnancy. 

Foundational studies of established critical ovarian / ovulatory factors have observed a similar trapped oocyte phenotype, sometimes with “normal” luteinization and sometimes with disrupted differentiation of the mGC layer [21]. Indeed, loss of CEBP/β, expression reduced ovulation in response to gonadotropins [29]. The present study similarly describes a delay that does not result in absolute infertility, illustrated by the gc*Adar*KO dam that produced two smaller litters of pups and the recovery of a small number of eggs from the oviducts of immature superovulated gc*Adar*KO mice, akin to what is seen in CEBP/β knockout mice. *COX-2*^−/−^ females are also infertile due to defective ovulation and fertilization [30]. The importance of timely expression of *COX-2* is highlighted in mice lacking the transcription factor CEBP/β. 

Ovulation itself is often explained as an inflammatory process [31]. The ovulatory surge of LH induces many inflammation mediators within the ovary including: leukocyte infiltration, edema swelling, tissue digestion and repair [21]. The ovary is a unique tissue that requires a delicate balance in inflammation to allow for ovulation and yet not so much that it impacts fertility. In contrast to hormonal control of mGC function, the extent of inflammatory cues and curtails have not been as well defined. A recent study displayed long term effects of manipulating the inflammasome within the ovary by suppression of *NLRP3* resulting in an improved reproductive rate at 12 months of age [32]. Interestingly, lack of *Adar* induces inflammatory pathways in many tissues [7,16,33,34]. Thus, it is possible that an increase in inflammatory responses in the mural granulosa *Adar* knockouts could be altering the delicate balance needed, ultimately preventing normal temporal sequences essential for proper ovulation. 

RNA seq analysis of GCs from gc*Adar*KO mice revealed upregulation of several inflammatory pathway genes including interferon induced protein with tetratricopeptide repeats 1 (*IFIT1*) which is reported to be a negative regulator of inflammatory genes [35]. Likewise, *IFIT3b* was upregulated in gc*Adar*KO after hCG in mGCs and this protein acts by binding to IFIT1 to extend its half-life, thereby regulating its specificity and activity [36]. It is possible that the delay in ovulation and presence of trapped oocytes at 16 h after hCG as observed in gc*Adar*KO could be due to blockade of the inflammatory response caused by IFIT1 and IFIT3B. It is well established that LH induces the expression of interferon-alpha (IFN-α), which is involved in the differentiation of preovulatory follicles [37]. Moreover, IFNα or IFNβ alone can induce the cumulus cell expansion and thereby ovulation [38]. IFI44 similar to IFIT1 and IFIT3B is another negative regulator of IFNα and β response and it was also upregulated [39]. Thus, upregulation of three negative regulators of the interferon pathway, prior to the LH surge, are thus likely blocking the positive LH induced IFNα and β effects on cumulus expansion, differentiation and ultimately ovulation.

Loss of *Adar* in other tissues has been shown to induce fibrosis in addition to inflammation [17]. Our initial analyses of the mating trial animals suggested that loss of ADAR1 might also be increasing ovarian fibrosis as collagen content was increased in the gc*Adar*KO mice. However, in a subsequent matched study of virgin WT and gc*Adar*KO mice from 2 to 16 months of age indicated that this change was likely due to a reduction in ovarian collagen content in the pregnant WT mice that experienced consecutive and almost continuous pregnancy compared to the infertile gc*Adar*KO mice. Similarly, we likewise did not observe a marked change in collagen deposition (i.e., PSR staining) between 2-16 months of age in the WT mice similar to prior studies [40], where the first evidence was observed at 22 months of age. It remains an interesting phenomena that pregnancy impacts ovarian collagen deposition, as well as the fact that *Adar* loss did not impact ovarian stromal fibrosis and for this reason we thought it was worth presenting the results of these two experiments. It is also worth mentioning that one aspect that differs between other tissues where ADAR1 is depleted, and our gc*Adar*KO is the ephemeral nature of the follicle and the subsequent corpus luteum. At the conclusion of the described breeding trial, presence of eosinophil structures, increased macrophage presence was also observed in the gc*Adar*KO, interestingly increased eosinophil abundance was reported to play a regulatory role in corpus luteum formation [41], and it is noted that luteal structures were not widely observed in the gc*Adar*KO mice. 

In addition to the inflammatory genes altered in the gc*Adar*KO mice, several other gene families showed marked changes. The oligoadenylate synthetase like protein family of genes consists of three classes in humans. They are OAS1, OAS2, OAS3 [42]. In mice eight subtypes of genes, i.e., *Oas1a*, *Oas1b*, *Oas1c*, *Oas1d*, *Oas1e*, *Oas1f*, *Oas1g*, and *Oas1h* are found to be homologues to OAS1 in humans [43]. Among these eight subtypes OAS1A and OAS1G alone have the ability to bind dsRNA and convert ATP into 2’, 5’ linked oligomers of adenosine (2-5A) [44,45]. OAS1D was reported to interact with OAS1A and inhibit OAS1A activity and block IFN/OAS/RNAsL-mediated RNA degradation in oocytes. *Oas1d* null mice show reduced fertility due to the lack of this protective effect [46]. We observed *Adar* plays a similar role and regulates the expression of *Oas1g*. Thus, lack of *Adar* caused higher expression of *Oas1g*, which in turn could mediate RNA degradation in oocytes and subsequently reduce fertility. A significantly down regulated gene following loss of *Adar* in granulosa cells, oviduct specific glycoprotein 1 (OVGP1) binds to the zona pellucida and helps the zona pellucida in acquiring resistance to proteolytic digestion during oviductal transit [47,48,49]. Its reduced expression by granulosa cells prior to ovulation could compromise the oocyte and oocyte-cumulus interaction and ultimately impact events such as cumulus expansion and oocyte maturation, which are vital for ovulation. 

## 4. Materials and Methods

### 4.1. Animals and Generation of Conditional Knockout

All procedures involving animals were reviewed and approved by the Institutional Animal Care and Use Committee at the University of Kansas Medical Center and were performed in accordance with the Guiding Principles for the Care and Use of Laboratory Animals. Experiments were performed on conditional knockout female mice, *Adar ^flox/flox^ Cyp19a1-*Cre/+ (gc*Adar*KO) and littermate controls (i.e., *Adar^flox/flox^* ^or *flox/+*^ lacking the *Cyp19a1-*Cre transgene hereafter collectively referred to as wild-type (WT)) generated by breeding homozygous *Adar ^flox/flox^* female mice with heterozygous *Adar ^flox/+^ Cyp19a1-*Cre/+ males. *Adar ^flox/flox^* mice were obtained from Dr. Stuart H Orkin’s lab [50] and the *Cyp19a1*-Cre mice from Dr. Jan A. Gossen [51]. Mice were maintained in a humidity and temperature-controlled environment with a 14 h light, 10 h dark cycle (7 am to 9 pm) with ad libitum access to food and water.

### 4.2. Assessment of Fertility

Fertility was assessed by pairing female gc*Adar*KO mice (42 days of age, n = 5) as well as control littermates (WT) lacking *Cyp19a1*-Cre (n=6) with adult (7–8-week-old) wild-type C57BL/6J males of known fertility. All females were continually exposed to males for 7 months. Female mice were euthanized at the conclusion of the breeding trial and ovaries were dissected away from the uterus proximal to the uterotubal junction, rinsed in sterile PBS and placed in cold 4% paraformaldehyde (PFA).

Estrous cyclicity was examined by vaginal cytology in a group of sexually mature gc*Adar*KO and WT 42-day old female mice in the absence of males for at least 21 days. Additionally, at ~5 months of the breeding trial, the vaginal cytology of the gc*Adar*KO females in the presence of fertile males was examined for 26 days. Vaginal cytology for control females in the breeding trial was not conducted due to continuous pregnancy. All cycle staging was performed at 8:00-10:00 a.m. and approximately 200 µl of sterile saline was gently flushed into and aspirated from vaginal opening using a glass dropper. The aspirated lavage was then placed on glass slides and allowed to dry at room temperature. Smears were stained with 1% crystal violet in water (*w*/*v*) for 2 minutes, followed by gentle rinsing with water. Samples were mounted with 15 µl of glycerol and coverslip for microscopic visualization on a Nikon YS2-T microscope at 10× magnification. Presence and relative abundance of leukocytes, nucleated epithelial cells, and cornified squamous epithelial cells were used to categorize mice into proestrus, estrus, metestrus, and diestrus [52]. Presence of sperm or visualization of a seminal plug preventing lavage in breeding trial females was recorded as a positive mating. 

### 4.3. Induction of Superovulation by Gonadotropins

Superovulation was induced in 21-day old mice via an intraperitoneal (i.p.) injection of 5 IU of equine chorionic gonadotropin (eCG; Lee Biosolutions, Maryland Heights, MO, USA) followed by 5 IU of human chorionic gonadotropin (hCG) 46–48 h later with relevant lengths of hCG (Millipore Sigma, St. Louis, MO, USA) exposure described for each experiment. To determine ovulation rates, mice were euthanized at 16, 18, and 20 h post-hCG administration and ovaries and oviducts were collected by dissection distal to the uterotubal junction to ensure complete removal of the oviduct without disruption. Typically, ovulation in mice occurs between 12–15 h post-hCG, thus the standard is to collect at 16 h post-hCG for assessment of ovulation rate [53]. In this study an additional 18 and 20 h post-hCG collection were included to determine if ADAR1 was temporally affecting the ovulatory process. The oviduct and bursal encapsulated ovary were washed in dPBS before separation in warmed FHM media (Millipore Sigma) supplemented with bovine serum albumin (BSA; 4 mg/ml, Millipore Sigma) in 35 mm dishes. Cumulus oocyte complexes (COCs) were expressed from oviducts and transferred to 25 µl FHM media droplets containing 5 µL of hyaluronidase (Millipore Sigma) at 10 mg/mL for ten minutes to remove cumulus cells. Freed oocytes were then counted, morphologically evaluated and then discarded. 

### 4.4. Histology

Ovaries were prepared for histological analysis by immediately placing them into cold 4% paraformaldehyde fixative. Fixation occurred overnight (~16 h) at 4 °C before ovaries were transferred to 70% ethanol prior to dehydration and paraffin embedding. Tissues were sectioned (Leica RM2035) at 7 µm thickness, placed on ProbeOn Plus slides in preparation for hematoxylin and eosin or picrosirius red (PSR) staining for visualization of collagen deposition [40]. PSR intensity was quantified using FIJI/ImageJ (NIH). 

PSR staining of aged (2, 6, 9, 12 and 16 m old) virgin gc*Adar*KO (n=5, 6, 5, 5, 3) and WT (n=3, 4, 3, 3, 3) ovary sections were imaged at 20x using brightfield microscopy (Nikon 80i). A sampling of three random regions of the ovaries were imaged and uploaded to the ImageJ software. Non-tissue space was cut out using the selection tool. Scale calibration, image gray scaling, and measurement of positive staining were done following NIH ImageJ PSR staining analysis protocol [54,55]. Threshold for staining intensity was maintained across all samples. 

### 4.5. Isolation of Mural Granulosa Cell RNA

Immature (21 d old) and mature (6–8-week-old) WT and gc*Adar*KO mice were superovulated as described above, at specific time points post-hCG administration mice were euthanized, and the ovaries were dissected free of the ovarian bursa and then rinsed in sterile dPBS to remove blood and tissue debris. Ovaries were then placed in dishes of FHM with 4 mg/ml BSA. Large antral follicles visualized under a Nikon SMZ1000 with a Nikon NI-150 high intensity illuminator were individually punctured with insulin syringes (28 gauge) to allow expulsion of mGCs and cumulus oocyte complexes. Smaller follicles were left with the remaining ovarian tissue which was removed from the dish before naked oocytes and cumulus oocyte complexes were removed and discarded. Mural GCs and the surrounding media was then collected, placed into a 1.5 ml Eppendorf tube and gently pelletized at 800× *g*, excess media was aspirated using a pipette, and 500 µL of TRI Reagent (Invitrogen, Waltham, MA) was applied to the cell pellet. Solubilized cell pellets were frozen at −80 °C overnight followed by completion of the manufacturer’s RNA extraction protocol (Invitrogen).

### 4.6. Quantitative RT-PCR Analysis and RNA Sequencing

Extracted total RNA was subjected to RNA quality analysis using an Agilent 2100 Bioanalyzer (Santa Clara, CA, USA) and samples with RIN values over 9 were selected for RNA sequencing, samples with RIN values above 8 were used for qRT-PCR analyses. RNA seq was performed on WT and gc*Adar*KO mGCs collected after eCG only injection (0 h) and 4 h post-hCG (n = 3 for each sample), while qRT-PCR was evaluated over a broader range of time points (0, 4, 8, and 12 h post-hCG; n = 6 for each sample). cDNA library preparation and sequencing were performed by KUMC Genomic Core. RNA sequencing was performed on Illumina NovaSeq 6000 sequencing machine (Illumina, San Diego, CA, USA). Sequencing generated between 28 to 32 million reads per samples. The read quality was assessed using the fastQC software. Sequenced reads were mapped to mouse genome (GRCm38 re197) using STAT software. Expression normalization and differential gene expression (DEG) were calculated using the DESeq2 (version 1.26.0). All RNA seq files are available at Sequence Read Archive portal (Bio Project: PRJNA794072 and SRR17423715-SRR17423728). Superscript II reverse transcription was used for the cDNA preparation, followed by qRT-PCR on a Applied Biosystems-Quant Studio 7 Flex using Power SYBR green master mix. Primers used in the present study are listed in Table 1. To normalize gene expression *Rn18S* was used in the comparative 2^-∆∆CT^ method.

### 4.7. Pathway Analyses

Using the Meta-chart software, two Venn diagrams were generated, (1) by overlapping DEGs of 0 h WT and gc*Adar*KO and 4 h WT and gc*Adar*KO granulosa cell RNA seq results, (2) by overlapping DEGs of 0 and 4 h WT with 0 and 4 h gc*Adar*KO mGCs results. The resultant DEG from these groups were subjected to Ingenuity Pathway Analyses to determine the functional pathways.

### 4.8. Statistics

Statistical analysis was performed using GraphPad Prism (Version 9 GraphPad Software Inc. San Diego, CA, USA). *T*-tests were performed to determine significance of PSR staining intensity (breeding trial animals) and litter size between WT and gc*Adar*KO groups. ANOVA was used to determine the significance of the number of eggs retrieved following superovulation and for the PSR staining time course from aged females. Quantitative RT-PCR results were analyzed by ANOVA followed by Duncan’s post-hoc mean separation tests. *p* value < 0.05 was considered significant.

## 5. Conclusions

In conclusion granulosa specific *Adar* knockout mice have relatively normal folliculogenesis but failed to respond appropriately to the ovulatory surge of LH/hCG resulting abnormal estrous cyclicity, failure to ovulate at the normal time (i.e., prior to 16 h post-hCG) and infertility. RNA seq analysis revealed upregulation of a subset of genes which includes *Ifit1, Ifit3b, Ifi44, Oas1g*, which are involved in the interferon and inflammatory pathways, consistent with ADAR1′s role in preventing dsRNA mediated interferon activation. The lack of *Adar*, thus lead to an altered inflammatory and interferon response within the granulosa cells, delaying ovulation and oocyte maturation.

## Figures and Tables

**Table 1:** List of primers used in the qPCR assays.

Symbol	Reference mRNA	Forward Primer and Reverse Primers	Amplicon
*Ifi44*	NM_001370771.1	F: CCAACTGACTGCTCGCAATA R: AATAGGACCCAGCAGCAGAA	190 bp
*Ifit1*	NM_008331.3	F:ATGGGAGAGAATGCTGATGG R: AGGAACTGGACCTGCTCTGA	137 bp
*Ifit3b*	NM_001005858.3	F: CCATGTTCCGCCTAGAAGAA R: TCTCCCATCCTCAGCAGTTT	130 bp
*Oas1g*	NM_011852.3	F:GCTGGGAGACCCAGGAAG R: GCACCTTGGAAGCATCTCTC	180 bp
*Ovgp1*	NM_007696.2	F: TTGTGGCCAAGAATCTGCAG R: GCAAGTGTGGAGAGCATAGC	148 bp
*Rn18s*	NR_046237.1	F:GCAATTATTCCCCATGAACG R: GGCCTCACTAAACCATCCAA	123 bp

## Data Availability

All RNA seq files are available at Sequence Read Archive portal https://www.ncbi.nlm.nih.gov/sra (Bio Project: PRJNA794072 and SRR17423715-SRR17423728).

## Supplementary Materials

The following supporting information can be downloaded at: https://www.mdpi.com/article/10.3390/ijms232214001/s1.
