# Supplementary figures and images for: Granulosa Cell Specific Loss of Adar in Mice Delays Ovulation, Oocyte Maturation and Leads to Infertility
Source: Int J Mol Sci. 2022 Nov 13;23(22):14001. doi: 10.3390/ijms232214001 (PMC9695778; doi:10.3390/ijms232214001)

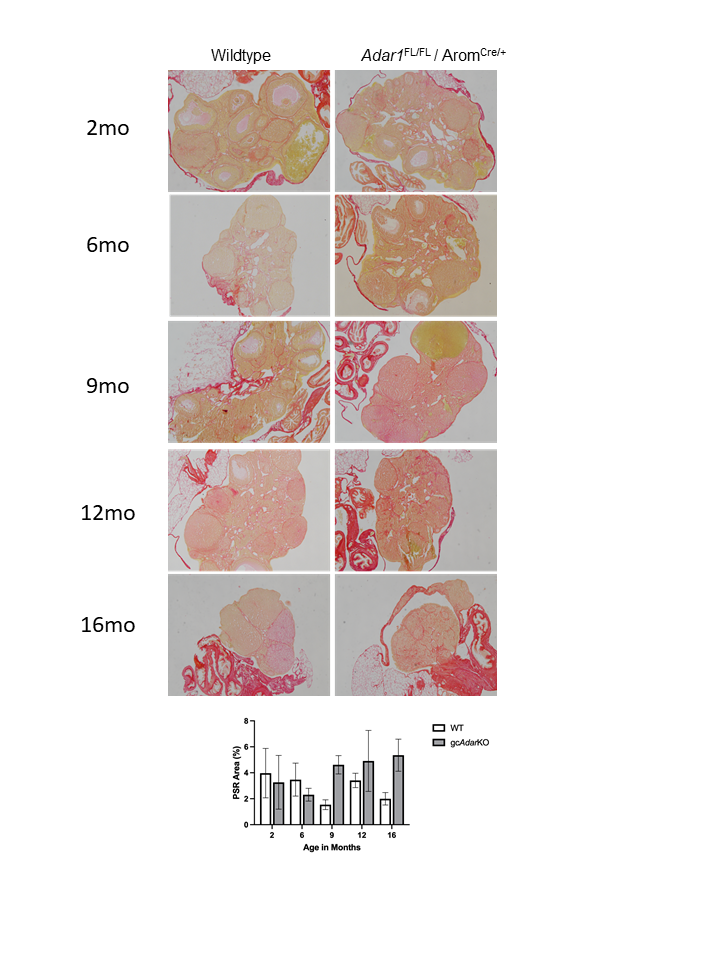

Supplement: Supplementary file 1 [file ijms-23-14001-s001.zip › Supplementary Figure S1.tif]
